# Reinterpretation of evidence advanced for *neo-*oogenesis in mammals, in terms of a finite oocyte reserve

**DOI:** 10.1186/1757-2215-4-1

**Published:** 2011-01-06

**Authors:** Elena Notarianni

**Affiliations:** 1 Department of Biological & Biomedical Sciences, Durham University, South Road, Durham DH1 3LE, UK

## Abstract

The central tenet of ovarian biology, that the oocyte reserve in adult female mammals is finite, has been challenged over recent years by proponents of *neo*-oogenesis, who claim that germline stem cells exist in the ovarian surface epithelium or the bone marrow. Currently opinion is divided over these claims, and further scrutiny of the evidence advanced in support of the *neo-*oogenesis hypothesis is warranted - especially in view of the enormous implications for female fertility and health. This article contributes arguments against the hypothesis, providing alternative explanations for key observations, based on published data. Specifically, DNA synthesis in germ cells in the postnatal mouse ovary is attributed to mitochondrial genome replication, and to DNA repair in oocytes lagging in meiotic progression. Lines purported to consist of germline stem cells are identified as ovarian epithelium or as oogonia, from which cultures have been derived previously. Effects of ovotoxic treatments are found to negate claims for the existence of germline stem cells. And arguments are presented for the misidentification of ovarian somatic cells as *de novo *oocytes. These clarifications, if correct, undermine the concept that germline stem cells supplement the oocyte quota in the postnatal ovary; and instead comply with the theory of a fixed, unregenerated reserve. It is proposed that acceptance of the *neo-*oogenesis hypothesis is erroneous, and may effectively impede research in areas of ovarian biology. To illustrate, a novel explanation that is consistent with orthodox theory is provided for the observed restoration of fertility in chemotherapy-treated female mice following bone marrow transplantation, otherwise interpreted by proponents of *neo*-oogenesis as involving stimulation of endogenous germline stem cells. Instead, it is proposed that the chemotherapeutic regimens induce autoimmunity to ovarian antigens, and that the haematopoietic chimaerism produced by bone marrow transplantation circumvents activation of an autoreactive response, thereby rescuing ovarian function. The suggested mechanism draws from animal models of autoimmune ovarian disease, which implicate dysregulation of T cell regulatory function; and from a surmised role for follicular apoptosis in the provision of ovarian autoantigens, to sustain self-tolerance during homeostasis. This interpretation has direct implications for fertility preservation in women undergoing chemotherapy.

## 1. Introduction

Since the mid-twentieth century, the prevailing principle in mammalian oocyte biology has been that female reproductive capacity is defined absolutely by the number and quality of primordial follicles having developed in the ovary by the neonatal period [1]. Acceptance of this principle was predicated on empirical evidence: that the mechanism of oocyte formation entails expansion from a relatively small population of primordial germ cells (PGC) in the foetal period, to provide a massive reserve of primordial follicles at birth [2,3]; and that gradual depletion of that reserve in the adult by atresia and ovulation leads to reproductive senescence and cessation or, specifically in humans, the menopause [4]. The predicted and observed consequence of this theory is that oocytes ovulated later in the reproductive period are of inherently poorer quality due to cellular defects, chromosomal abnormalities and functional deteriorations that accumulate with age [5,6].

Recent years have seen repeated challenges to this orthodoxy, constituting a revival of the concept of *de novo *oogenesis in the adult ovary, or *neo-*oogenesis. The key studies and ensuing discourse are summarised as follows. Diverse groups have purported evidence for *neo*-oogenesis in mice, from germline stem cells existing specifically in the ovarian epithelium [7-11]. Moreover, claims were made that female germline stem cells originate at a site extraneous to the ovary, namely the bone marrow, and are transported to the ovary *via *the circulatory system [12,13]: a scenario that would represent a radical transformation of the established theory of germline specification [2,3]. The study of Eggan *et al. *[14], using parabiosis between female mice to demonstrate that ovulated oocytes are not derived from transfused precursors, is significant in countermanding claims for the provision of oocytes *via *the circulation [12]. But this was in turn refuted by Tilly *et al. *[15], who deduced evidence for crossengraftment of oocytes supplied from a parabiont, in a robust defense of the *neo-*oogenesis concept. Abban and Johnson [16] find further support for *neo-*oogenesis in the derivation of so-called "female germline stem cell" (FGSC) lines by Zou *et al. *[10]. Pacchiarotti *et al. *[11] also claim the establishment of ovarian germline stem cell lines, and endorse the *neo*-oogenesis hypothesis. Meanwhile, cogent arguments were made against the replenishment of oocytes, from statistical analysis of the follicle pool over the reproductive period in mice [17,18]; and a recent study involving mathematical modelling of the ovarian reserve found no evidence to support the occurrence of *neo*-oogenesis in humans [19].

To date, a consensus has yet to emerge regarding the validity of *neo*-oogenesis in relation to adult female mammals, and forthright opinions have been expressed in favour of [13,15,16,20] and against [14,17,21-24] the hypothesis. Furthermore, qualified support has been expressed for the occurrence of *neo*-oogenesis in mice, but not in humans [19]. In another permutation of the hypothesis, germline stem cells exist in adult mouse ovaries but are quiescent under physiological conditions [25], functionally contributing to the oocyte reserve only in response to ovotoxic damage [26].

Thus, the debate continues and a consensus has yet to emerge. Further scrutiny of the evidence advanced in support of the *neo-*oogenesis hypothesis therefore is warranted - particularly in view of the enormous implications it holds for female fertility and health. Moreover, establishing the mechanism of oocyte allocation is fundamentally important to developmental, comparative and reproductive biology. This article contributes arguments against *neo*-oogenesis, revisiting underlying assumptions and providing alternative explanations (summarised in Table 1) for observations advanced - and maintained - as key by advocates of the hypothesis, adding to the considerable body of criticisms already levied. If the *neo-*oogenesis hypothesis is incorrect, an alternative explanation is required for a significant finding made by its proponents: the restoration of fertility by bone marrow transplantation (BMT) to chemotherapy (CT) treated mice.

**Table 1:** Key observations advanced in support of *neo*-oogenesis in mammals, and proposed alternative explanations.

Section	Observation	Interpretation by proponents of *neo-*oogenesis	Alternative explanation consistent with a fixed **oocyte reserve**.
**2**.**(i)**	BrdU-incorporation in Mvh^+ ^germ cells located in the OSE [7,10].	Mitosis in germline stem cells.	MtDNA synthesis, and DNA recombination and repair in tardy oocytes, in the neonatal ovary.
	Mvh^+ ^germ cells located in the OSE [7-9].	Existence of a germinal epithelium.	Oocytes in transit across the OSE during exfoliation [54].
			
**2**.**(ii)**	"Oocyte-like" phenotype of cells in OSE-derived cultures [8,9].	*De novo *formation of immature and secondary ocytes from stem cells.	Nondescript cells undergoing oncosis.
	Small, round cells, above and below the OSE [9].	Putative female germline stem cells.	Small immune cells in the OSE [54].
	"Embryoid body-like" and "blastocyst-like" structures [9] in OSE-derived cultures.	Pathenogenetic activation of *de novo *oocytes.	Nondescript cellular aggregates, and vesicles of OSE.
	Expression of *Oct4, Sox2, Nanog *and *c-kit *by OSE derivatives [9].	Embryonic-like, germline stem cells.	Cultures containing regenerative epithelium [58].
	Cell lines producing early oocytes [11].	Female germline stem cell lines.	Mixed cultures of OSE, early oocytes and/or oogonia.
			
**2**.**(iii)**	BU-induced depletion of the follicle pool [7,15] and extinction of fertility.	Destruction of replicative, female germline stem cells by BU treatment, without atresia.	Induction of oocyte atresia by BU treatment; and proof of absence of female germline stem cells.
			
**2**.**(iv)**	EGFP^+ ^cells with germ-cell markers in ovaries of CT-treated mice following BMT or PBCT [12,13].	*De novo *oocytes from bone marrow-derived precursors.	*Oct4*-expressing macrophages; and autofluorescent, somatic cells of the ovary.
	Presence of PGC and HSC in extraembryonic regions during early post-implantation development [12].	Incorporation of oocyte precursors within the haematopoietic system.	Distinct temporal and spatial niches for the origins and migration of germinal and haematopoietic lineages.
			
**2**.**(v)**	Replicative, unipotent oocyte-like cells [10].	Existence of female germline stem cells.	Residual oogonia induced to proliferate by specified culture conditions, and expansion of populations of functional oogonia.
	Immuno-magnetic isolation of Mvh^+^ proliferating cells from disaggregated ovaries [10].	Selective purification of stem cells *via *Mvh binding to anti-Mvh antibody.	Harvesting of oogonia and primary oocytes due to Mvh binding to anti-Mvh antibody, or to Fc receptors on the plasma membrane of oogonia and oocytes binding to Fc moiety of antibody.
			
**3**.	Restoration of the host follicle pool in CT-treated mice following BMT [12,13].	Stimulation of endogeneous, *de novo *oogenesis.	Induction of autoimmunity to ovarian antigens by CT; and rescue of fertility *via *tolerance restored by haematopoietic chimaerism.

## 2. Evidence advanced for *neo-*oogenesis

### (i) BrdU-incorporation by germ cells located in the ovarian surface epithelium

A primary observation made in mice by proponents of *neo-*oogenesis has been the incorporation of the thymidine analogue, 5-bromo-2-deoxyuridine (BrdU), by germ cells located in the ovarian surface epithelium (OSE), as detected by immunocytochemistry using anti-BrdU monoclonal antibody: this was interpreted as evidence for mitotic germ cells [7,10], with the OSE functioning as a classical, germinal epithelium [7-9,11]. Johnson *et al. *[7] discounted the alternative possibilities that BrdU-incorporation arose from either mitochondrial (mt) DNA replication or DNA repair in oocytes, on the basis that "the degree of BrdU incorporation observed in cells due to either of these processes is several log orders less than that seen during replication of the nuclear genome during mitosis." This assumption is invalid because the immunocytochemical technique used is both likely and sensitive enough to detect **(a) **mtDNA synthesis and **(b) **DNA repair in meiotically arrested oocytes, as discussed below.

#### (a) Anti-BrdU antibody detection of mtDNA synthesis

In studies using anti-BrdU immunocytochemistry to observe cell proliferation, BrdU incorporation into mtDNA may be discounted where mtDNA constitutes a minor fraction of total cellular DNA (< 0.2% in the case of L cells, or 50 mtDNA molecules per cell [27]). Here, anti-BrdU antibody is saturated by binding to BrdU-substituted nuclear DNA (nDNA), and the relatively much lower incorporation of BrdU into mtDNA goes undetected [28]. However, early studies established that mtDNA replication occurs autonomously to that of nDNA in cultured cells; and that in the absence of nDNA replication, mtDNA can be labelled with BrdU to a high specific activity [29,30] that is detectable by anti-BrdU immunocytochemistry, with short incorporation periods (1-2 h) commensurate with mtDNA replication times [28]. It is therefore argued that for mammalian oocytes in particular, mtDNA synthesis would be readily detectable: not only is nDNA replication absent, but also the number of mitochondria is considerable, increasing from <200 in PGC to ~6,000 in the resting oocyte of the primordial follicle [31]. The mouse secondary oocyte contains ~92,000 mtDNA copies [32]. Hence, it is feasible that the aforementioned studies of Johnson *et al. *[7] and Zou *et al. *[10] would have detected *in situ *mtDNA incorporation in prophase-arrested oocytes.

This deduction is supported in both studies [7,10] by the apparent co-localisation of immunofluorescence for BrdU with mouse VASA-homologue (Mvh), the germ cell-specific protein that is cytoplasmic in location [33]. For example, in the report of Johnson *et al. *[7], Figure two 'd' shows a clearly defined oocyte at the ovarian surface stained with anti-BrdU immunofluorescence (red signal) co-localised with anti-Mvh immunofluorescence (green signal) to give a strong, combined yellow signal dispersed throughout the cytoplasm. (In cultured cells [28] and oocytes [34], newly synthesised mtDNA is initially located at a perinuclear location, adjacent to the nuclear boundary, and becomes dispersed in the periphery of the cell with time.) If, as claimed by Johnson *et al. *[7], BrdU incorporation represented nDNA replication, this would require the cell to have attained prometaphase (at which stage the nuclear membrane breaks down) so that BrdU incorporation would be detectable in the cytoplasm. However, it is highly unlikely that during the 1 h labelling period the cell could have exited S-phase and transited G_2 _and prophase, and so nuclear DNA replication can be discounted. In the report of Zou *et al. *[10], Figure S1 shows nuclear staining for anti-BrdU immunofluorescence (green signal) in the nuclei of primary oocytes in 'a', but also co-localisation with anti-Mvh immunofluorescence (red signal) to give a yellow signal in 'a', 'b', 'd' and 'e'. Moreover in 'a', the yellow signal is closely juxtaposed to the nuclear boundary, in keeping with mtDNA synthesis at this location occurring simultaneously with nuclear incorporation. To summarise, it is inferred that the examples of BrdU-labelled germ cells presented by Johnson *et al. *[7] and Zou *et al. *[10] provide direct evidence for mtDNA synthesis occurring in oocytes located at the surface of the neonatal [7,10] and adult [10] mouse ovaries.

#### (b) Anti-BrdU antibody detection of DNA recombination and repair

The condition allowing detection of mtDNA synthesis by *in situ *BrdU immunocytochemistry, namely an absence of nDNA replication [28], would also allow detection of nDNA synthesis arising from recombination and repair by the same technique. Accordingly, *in situ *BrdU immunocytochemistry has been used to reveal DNA repair in mammalian cells [35]. And the detection of stretches of single-stranded BrdU-substituted DNA at sites of meiotic recombination in mouse spermatocytes illustrates the sensitivity of this method [36].

In mammals, the meiotically arrested oocyte contains the enzymatic capacity for DNA repair pathways [37], and circumstantial evidence for this activity was obtained by Oktay *et al. *[38] from expression of the DNA-repair associated protein, PCNA, in growing and atretic rat oocytes. Although the extent of DNA synthetic activity arising from DNA recombination and repair in oocytes at earlier stages is unclear, it may not be negligible. The meiotic process in the oocyte is highly error prone [39], which leads to high rates of elimination of immature oocytes, especially at diplotene in the neonatal period [40]. Meiotic recombination occurs during the pachytene stage of prophase I, prior to diplotene arrest; and in the mouse this latter stage is reached by most oocytes by day 5 postnatal [41]. As meiotic prophase I is asynchronous, the temporal window for meiotic recombination extends into the neonatal period: non-apoptotic, pre-diplotene (zygotene and pachytene) oocytes have been noted to persist for at least 2 d after birth, with 7.4% of oocytes in pachytene on day 2 postnatal [40]. This is a most relevant finding, which was attributed by Ghafari and colleagues [40] to a prolongation of early stages of meiosis in a proportion of oocytes, necessitated by ongoing DNA recombination or repair. By inference, such a population of pre-diplotene stage oocytes engaged in recombination or repair activities would be readily detectable by *in situ *BrdU immunocytochemistry, in the neonatal mouse ovary. The distinct, nuclear staining for BrdU in the oocyte of Figure two 'e' of Johnson *et al. *[7], and in oocytes in Figure S1 ('a') of Zou *et al. *[10], could therefore be attributed to DNA recombination or repair.

In summary, the immunofluorescent detection of BrdU incorporation into oocytes of the neonatal mouse [7,10] can be ascribed to mtDNA synthesis where BrdU incorporation is cytoplasmic, and to DNA recombination and repair where incorporation is nuclear, rather than to replicative nDNA synthesis alone. These alternative explanations may be relevant also to the detection of thymidine incorporation in diplotene and atretic oocytes in the ovaries of adult prosimian primates [42,43]. Crone and Peters [44] previously documented the incorporation of tritiated thymidine into the nuclei of early diplotene oocytes of mice injected in the neonatal period. These labelled oocytes were in nascent follicles located centrally in the ovary, and were cleared within a few days. The authors considered the phenomenon most likely represented abnormal DNA synthesis and repair in degenerating oocytes, whose frequency may have been underestimated owing to the lack of sensitivity of their technique. These considerations provoke the question, what is the reason for the location of BrdU-labelled oocytes in OSE [7,10]? Perhaps these studies present a snapshot in a poorly understood process contributing to oocyte attrition in both mouse and human - the extrusion of oocytes from the ovarian surface and into the peritoneal cavity [24,45], which was postulated by Motta *et al. *[45] to occur beyond the neonatal period, to puberty. Could these surface oocytes be defective, as postulated by Crone and Peters [44]?

### (ii) Cultured OSE gives rise to "oocyte-like" cells

Following the deduced existence of mitotic germ cells in the OSE (above), Bukovsky *et al. *[8] and Virant-Klun *et al. *[9] endeavoured to culture OSE derivatives, and subsequently reported the production of "oocyte-like" cells *in vitro*. Two major limitations are common to both studies.

**(a) **The criteria used to denote an "oocyte-like" phenotype [8,9] are morphological, namely: cells with large and rounded morphology in which a large or no nucleus is visible, and which may be surrounded by a structure resembling a zona pellucida (ZP). However, the photomicrographs presented may instead depict those general features of cells undergoing apoptosis, necrosis or - especially - oncosis [46], namely: cell swelling, plasma membrane breakdown, and swollen or lysed nuclei. Structures described as "developing zona pellucida" [8,9] may reflect cellular swelling, membrane rupture and lysis, and spillage of cytoplasm [46]; the "germinal vesicle" [8,9], nuclear swelling [46]; and "germinal vesicle breakdown" [8,9], karyolysis [46]. These considerations underline the importance of validating putative oocytes by immunocytochemical and molecular techniques, rather than by morphological criteria. The attempt by Bukovsky *et al. *[8] to detect ZP-antigenicity in these cells by immunofluorescence is marred throughout by a high background of staining of the cytoskeleton, which is probably an artefact of desiccation arising from the unconventional step of air-drying cells overnight, prior to fixation. Desiccation and cell death occur extremely rapidly under these conditions [47,48], with interim activation of survival and death pathways [49]. Regarding the deduced ZP-antigenicity of OSE-derived "germ-like" cells as detected using PS1 antibody [8], it should be noted that Skinner and Dunbar [50] considered their antibody to be non-specific for ZP proteins as it recognises a carbohydrate moiety present on the apical surface of the OSE.

**(b) **It is immediately apparent that the culture systems of Bukovsky *et al. *[8] and Virant-Klun *et al. *[9] are relatively very simple, without addition of the growth factors, cytokines or feeder-cell support that usually are essential to the growth of pluripotent germline cells or ES cells. In fact, the growth of embryonic or germline stem cells under these conditions would be unprecedented. What cells, therefore, could constitute the proliferating populations in these studies?

As cultures were obtained by the conventional technique of scraping of the OSE, the heterogeneity of cells should be considered: an estimated 98% of cells obtained in this way are ovarian epithelial cells [51], and contaminants include extraovarian mesothelial cells, endothelial cells, ovarian somatic and mesenchymal cells, and immune cells [52]. Moreover, cultured OSE demonstrates an epitheliomesenchymal phenotype with contractile functions, and the capacity to differentiate into stroma, granulosa cells or Müllerian epithelia, reflecting its role *in vivo *as a dynamic tissue involved in post-ovulatory tissue repair and remodelling [52]. Granulosa cells express *Oct4 *and are multipotent, differentiating into neurons, chondrocytes and osteoblasts [53]. Therefore, in the absence of data from clonal cell analysis, and of unambiguous validation by stem cell-specific markers (see below), the claims of Bukovsky *et al. *[8] and Virant-Klun *et al. *[9] for spontaneous *in vitro *differentiation of germline stem cells into cells of mixed phenotype should be regarded with caution.

The cell types cultured by Virant-Klun *et al. *[9] from OSE scrapings from postmenopausal women, termed "putative stem cells", "oocyte-like", or "embryonic", may be re-identified from information in the literature. "Putative stem cells" were identified morphologically as round cells, 2-4 μm in diameter, located below or above the OSE [9]. However, the possibility arises that these are small immune cells, e.g. lymphocytes or plasma cells, which are seen located above and below the OSE in ovarian sections [54]. After enrichment by differential centrifugation, these "putative stem cells" proliferated in culture [9]. Plasma cells, also, can be cultured easily in simple media [55], but the presence of this cell type as a culture contaminant was not considered [9]. Virant-Klun *et al. *[9] stated that the proliferating "putative stem cells" generated adherent oocyte-like cells, 20-95 μm in diameter, with ZP-like, germinal vesicle-like and polar body-like structures that were ascribed to an oocyte nature. However as stated above, these structures could arise from oncosis in any of the cell types being cultured, causing cell swelling, karyolysis and cytoplasmic leakage. In their cultures, Virant-Klun and colleagues [9] also describe the formation of "embryoid body-like" and "blastocyst-like" structures, interpreted as products of parthenogenetic activation of oocyte-like cells. However, they are far less convincing in appearance than the (parthenogenetic) embryos demonstrated by Hübner *et al. *[56] to arise from ES cell differentiation into oocytes. Could there be an alternative explanation for the structures produced by Virant-Klun *et al. *[9]? The aggregates of cells termed "embryoid-body like" could arise from any cell type, rather than being diagnostic of embryoid bodies proper with their complex internal differentiation. And the vesicles formed by these aggregates with continued culture could arise from a contaminating epithelial cell type, such as OSE [52], which has the capacity to polarise and form impermeable junctions. The propensity to form vesicles in culture is a common property of epithelial cells from epithelial linings [57]; and the increased tendency of OSE to line clefts and inclusion cysts in the ovary, with increasing age, may be relevant here [52]. Further clues to the identity of the cells can be gleaned from patterns of transcription: "putative stem cells" expressed *OCT4*, *SOX-2*, *NANOG *and *C-KIT*, and "blastocyst-like" structures expressed *OCT4*, *SOX-2 *and *NANOG*, from which an embryonic nature of the putative stem cells was inferred by Virant-Klun *et al *[9]. However, a recent study by Song *et al. *[58] first showed that the trio of stem cell regulatory genes, *Oct4*, *Sox-2 *and *Nanog*, constitute markers for epithelial stem cells, whose function is vital to regeneration and tissue homeostasis: they are expressed during the regeneration of rat tracheal epithelium *in vitro*, specifically by epithelial stem cells in the G_0 _phase. Expression of *Oct4 *is associated also with a variety of types of epithelial stem cells, but not their differentiated derivatives [59]. Moreover, human epithelial ovarian cancer cell lines and the multilayered structures, or spheroids, they form in suspension culture are known to highly express stem cell-specific genes, including *OCT4*, *NANOG *and *NESTIN *[60,61]. It is therefore inferred that the OSE-derivative cultures of Virant-Klun *et al. *[9] comprise epithelial stem cells, which are responsible normally for maintaining the integrity of the OSE - a property that may be especially important in ovaries of post-menopausal women [54], used here. This inherent regenerative potential may be manifest in culture. Another feature is consistent with the presence of OSE in these cultures - the expression of *C-KIT *[51]. In fact, both *C-KIT *and *KIT LIGAND *are expressed by human, normal OSE [62].

The importance of critically evaluating claims for the validation of cell lines as female (or ovarian) germline stem cells is further illustrated by the recent study of Pacchiarotti *et al. *[11]. These authors reported the isolation and characterisation of germline stem-cell lines from ovaries of neonatal mice of the TgOG2 strain. (These mice carry an *Oct4-GFP *transgene where *GFP *expression is controlled by an *Oct4 *promoter sequence. They are considered in more detail in section **2**.**(iv)**.) Their main conclusions are as follows:

**(a) **Germline stem cells were identified at the ovarian surface, on the basis of their small size (10-15 μm) and expression of *Oct4-GFP*, *Mvh*, *c-kit *and *SSEA-1*. These cells were purported to transition into germ cells of intermediate size (20-30 μm), and subsequently into growing oocytes.

**(b) **Cell populations containing the putative stem cells were isolated from disaggregated suspensions of whole ovaries by fluorescence-activated cell sorting for *Oct4-GFP *expression, and propagated using a feeder-based culture system. It was deduced that the derived lines consisted of ovarian germline stem cells from their expression of germ-cell and stem-cell markers (namely, Gcna1, c-kit, Oct4, Nanog and GFR-α1).

**(c) **Further evidence for the status of these cells as germline stem cells was presented from the formation of "embryoid bodies" containing differentiated derivatives of the three germ layers, mesoderm (denoted by expression of Bmp-4 and troponin), ectoderm (Sox-1, Ncam, nestin) and endoderm (FoxA2, Gata-4); and the production of early stage oocytes during culture.

However, many of these assumed marker specificities are incorrect and the above conclusions are therefore unwarranted, as discussed in detail below. Rather, it is proposed that the cultures consisted of monolayers of OSE, together with a proportion of early oocytes and/or oogonia. That is, a complex co-culture system is envisaged containing both somatic and germ-cell types. It is notable that the culture medium used by Pacchiarotti *et al. *[11] was optimised for spermatogonial stem cells (SSC) [63], as was that employed by Zou *et al. *[10] for FGSC. These media are considered further in section **2**.**(v)**, as potentially being mitogenic for growth-arrested oogonia.

**(a) **Rather than providing direct evidence for germline stem cells, the localisation of small cells (≤15 μm) expressing Oct4, Mvh and SSEA-1, and subtending the OSE, is compatible with residual oogonia [64-66]. In fact, the authors acknowledged the likely existence of oogonia in these neonatal ovaries.

**(b) **These putative germline stem cell lines show a striking resemblance in morphology and growth characteristics (with a low mitotic rate) to previously established mouse and human OSE cell lines [67-69], growing in monolayers as epithelial colonies with cobblestone appearance, with a tendency towards multilayering at the centre. (Compare, for example, the cellular morphology in Figure three 'N' of Pacchiarotti *et al. *[11] with that of mouse OSE in Figure two 'A' of Roby *et al. *[67] and in Figure four 'B' of Szotek *et al. *[69].) Like established lines of mouse OSE cells at low passage [67], these putative stem cells lacked tumorigenicity in mouse xenograft systems. Furthermore, markers reportedly expressed by these cultures are not germline specific: GFR-α1 is expressed by OSE [70]; and co-expression of *c-kit*, *Oct4 *and *Nanog *was discussed in section **2**.**(ii)**, in the context of the OSE as a regenerative epithelium.

**(c) **Concerning the structures described as "embryoid bodies", patterns of gene expression were entirely consistent with OSE, as a mesoderm-derived, multipotent epithelium with stromal characteristics. For example, nestin [60] and Gata-4 [69] are markers for OSE stem cells. FoxA2 is known to be expressed in uterine glands [71], and expression in this culture system may therefore be indicative of OSE cells undergoing Müllerian-type differentiation towards endometrioid cells [72]. In short, the structures described resemble those spheroids that are formed by both normal OSE [68,73] and ovarian cancer-derived cell lines [60].

Detection of Gcna-1 in these cell lines requires further comment, as this antigen is considered specific to the nuclei of germ cells in the neonatal and foetal gonad, from zygotene through pachytene stages of meiotic prophase. It is relevant that Alton and Taketo [74] observed immunocytochemical staining for Gcna1 in a large number of cells either in, or protruding from, the OSE in foetal mouse ovaries at 18.5 d.p.c., which was attributed to oocytes in the process of exfoliation. However, that those cells did not express Mvh [74] is incompatible with their identification as oocytes. It is therefore suggested that Gcna-1 may be expressed by OSE, especially during the neonatal period or in culture. Another germ cell-specific gene, *VASA*, is expressed by ovarian epithelial cancers, which arise from transformation of the OSE [75]. Now that candidate stem cells for OSE have been identified by Szotek *et al. *[69], it will be of interest to determine if genes involved in germ-cell specification also are involved in normal epithelial regeneration, or differentiation. As well as increasing understanding of the etiology of ovarian epithelial cancers, this information will help clarify the origin of cell lines claimed to represent ovarian germline stem cells [8,9,11] on the basis of expression of germ-cell markers.

### (iii) Busulphan-induced depletion of the follicle reserve

Recently, Tilly *et al. *[15] cited their findings from busulphan (BU) treatment of female mice as key evidence for *neo-*oogenesis, based on their understanding that this chemotherapeutic, alkylating agent targets replicative - and not postmeiotic - germ cells in females, as well as males. By their reasoning, inhibition of *de novo *oocyte formation by BU treatment leads to exhaustion of the oocyte reserve by normal processes during oestrus cycling: "Young adult female mice treated with busulfan exhibit a gradual loss of the entire primordial follicle reserve over a 3-wk period without a corresponding cytotoxic effect on primordial follicles [7]. Such an outcome would be expected if busulfan were, as past studies contend [76], selectively eliminating replicative germ cells that support primordial oocyte formation. The net result would be the normal rate of follicle loss *via *atresia no longer partially offset by *de novo *follicle formation, leading to accelerated depletion of the follicle reserve without the need for a corresponding increase in the rate of oocyte death." However the major premise here, that BU targets only replicative (and, by definition, premeiotic) germ cells in both females and males without causing atresia in postmeiotic cells (oocytes and spermatids), is seen to be incorrect from what is discussed below. Furthermore, it is deduced that the data of Johnson *et al. *[7] provide direct evidence *against neo*-oogenesis, and *against *precursors to oocytes being supplied from bone marrow precursors. To this end, it is necessary to consider the known effects of BU on female and male, murine reproductive function.

#### (a) BU causes atresia in oocytes and disrupts folliculogenesis

Although early studies in the rat established that BU-treatment during pregnancy induces lethality in the replicative oogonia of the foetus [77,78], substantial evidence indicates that the effects of BU are not confined to this stage. Burkl and Schiechl [79] observed that in the adult rat, chronic BU treatment is disruptive to the whole process of folliculogenesis: antral and secondary oocytes show diminished growth, with rapid and extensive degeneration; and younger follicles show abnormal development into distinct follicular structures with enlarged oocytes having only a single-cell layer of granulosa, correlating with late secondary or antral stages. These aberrant follicles were inferred to arise from inhibition of mitosis in the somatic cells, including granulosa cells. And in some of these single-layered structures, follicular fluid was seen to accumulate in a fissure-shaped antrum between the ZP and the follicular epithelium. (Such a hallmark of BU-induced ovotoxicity may be exemplified by the abnormal follicle in Figure four 'c' of Johnson *et al. *[7], to the upper left of the photomicrograph.) The work of Generoso *et al. *[80] informs of the gross effects on oocytes of a single administration of BU (or Myleran) in juvenile female mice: there is a dose-dependent, detrimental effect on fertility (at doses of 10-60 mg/kg i.p.) due to a progressive depletion of oocytes at the advanced as well as the earliest stages of development. Fertility is extinguished irreversibly after injection with 40 or 60 mg/kg; and at 40 mg/kg the total oocyte count diminished precipitously 7-14 d posttreatment.

In other words, and contrary to the claim by Johnson *et al. *[7] and Tilly *et al. *[15] that oocytes are refractory to the effects of BU, previous studies show that in the adult murine, BU exerts an immediate and lethal effect on late stage oocytes [79,80] that is accompanied by an aplasia resulting from active destruction of the primordial follicle pool [80].

#### (b) Predicted mechanism of BU cytotoxicity in folliculogenesis, *via *suppression of c-kit/SCF signaling

Further insight into the mechanism of action of BU can be gained from its effects on male germline stem cells (i.e. spermatogonial stem cells (SSC)) and on haematopoietic stem cells (HSC). Tilly *et al. *[15] stated that SSC are depleted by BU treatment. However, the work of Choi and colleagues [81,82] shows that the converse is true: SSC survive BU treatment in mice, while differentiating spermatogonia, meiotic spermatocytes and postmeiotic spermatids are depleted *via *apoptosis. A mechanism of action was deduced whereby BU induces loss of *c-kit *expression in these susceptible populations, with concomitant downregulation of c-kit/SCF signaling, leading to a block in G_1 _due to inhibition of PCNA synthesis. Meanwhile, the quiescent SSC are unaffected by BU due to their lack of *c-kit *expression, and spermatogenesis is fully restored eventually by these testis-repopulating cells [81]. In other words, abrogation of *c-kit *function is central to the mechanism of action of BU on spermatogenesis. By extension, we can infer significant consequences of BU-induced downregulation of c-kit/SCF signaling for folliculogenesis. Hutt *et al. *[83] review evidence from mouse models that the paracrine c-kit/SCF signaling pathway is crucial for activation of primordial follicles, oocyte survival and growth, and maintenance of meiotic arrest in small antral follicles. (This is in addition to roles in PGC colonisation of the ovary, proliferation of oogonia, proliferation of granulosa cells, and recruitment of thecal cells.) For humans also, there is evidence for paracrine and autocrine roles of this pathway in primordial follicle assembly and throughout folliculogenesis. Functional studies directly implicate c-kit in controlling folliculogenesis: antibody-induced blockade of c-kit causes attenuation of follicular development in neonatal and adult mice [84], and promotion of oocyte death *in vitro *[85]. Kissel *et al. *[86] documented arrested development of follicles in juvenile *c-kit *mutant mice, with mainly single-layered follicles predominating (*cf*. abnormal follicles of Burkl & Schiechl [79], described above). Therefore, functional c-kit is prerequisite to the survival and development of preovulatory follicles, and to granulosa cell proliferation. The documented effects of BU on developing and antral follicles [79] are now interpretable in terms of downregulation of c-kit/SCF signaling. The deduction of Yoshida *et al. *[84] is relevant, that in haematopoiesis, hair follicle melanogenesis, and spermatogenesis, c-kit function is required for differentiation and survival of cells that have advanced from stem cell pools, but not for the maintenance of quiescent stem cells. This is fully substantiated for spermatogenesis by the studies of Choi *et al. *[81], described above.

#### (c) BU induces transient myelosuppression with irreversible sterility

Lastly, in view of the bone marrow-derived oocyte precursors proposed by Johnson *et al. *[12], the effect of BU as a chemotherapeutic agent on haematopoiesis should be considered. Would BU treatment impinge on a precursor population from that source? The dose of BU used by Johnson *et al. *[12], namely 2 injections at 20 mg/kg i.p., 10 days apart, is not myeloablative but would cause transient myelosuppression, which is resolved in the strain used (C57BL/6) by 4-5 weeks [87]. (A myeloablative dose is 150 mg/kg [88].) For HSC, therefore, long-term repopulating stem cells would not be deleted by this BU dosage [89]. If oocytes are BM-derived, resumption of haematopoiesis should lead to restoration of fertility in BU-treated mice. However, fertility was extinguished in the studies of Johnson *et al. *[7], as it was also in the study of Generoso *et al. *[80] with similar BU dosages (see **(a)**, above). Therefore, the absence of restoration of fertility in BU-treated mice is taken as direct evidence against BM as a source of precursors for *neo*-oogenesis [7,12].

In summary, the data of Johnson *et al. *[7] on BU treatment of female mice causing aplasia and ovarian failure are interpretable entirely by cytotoxicity to early and late stage oocytes, and disruption of folliculogenesis. Evidence from other systems (spermatogenesis, haematopoiesis) implicates BU-induced down regulation of c-kit/SCF signaling, the function of which pathway is critical to folliculogenesis.

### (iv) Oocyte precursors from peripheral blood

Johnson *et al. *[12] modified their concept of *neo-*oogenesis to specify that oocyte progenitors are supplied to the ovary by the bone marrow *via *the circulatory system. This came from experiments on wild type (wt) and *Atm*-deficient (*Atm*^-/-^) mice in which sterile, depleted ovaries were reportedly repopulated with oocytes derived from EGFP-labelled progenitors, following peripheral blood cell transplantation (PBCT). Subsequently there have been other reports of successful engraftment of donor somatic cells as oocytes following CT and BMT [13], with the provisos that: only a low percentage of designated immature oocytes are donor-derived (around 0.1% of total oocytes in recipients) when bone marrow or peripheral-blood cells are transplanted; designated follicles are never observed beyond preantral stages (i.e. maturing antral or Graafian follicles); and donor cell-derived mouse offspring have never been produced. (Meanwhile, other attempts to reproduce these findings have proved entirely unsuccessful [14,23].) The general consensus is that any *de novo *follicles do not undergo ovulation, although they may support the depleted ovary [13]. What, therefore is the functional relevance of this proposed, renewing population of early-stage oocytes? Arguments leading to alternative identities for those cells designated as *de novo*, immature oocytes [12,13] are given below.

#### (a) Identification of *de novo *oocytes relies on germ-cell specificity of Oct4 expression

Attention is drawn here to the hypothesis of Eggan *et al. *[14] that bone marrow-derived cells might co-express germ cell-specific markers, and that the cells designated as immature oocytes by Johnson *et al. *[12] could have been misidentified. This hypothesis subsequently was refuted by Lee *et al. *[13] on the basis that expression of the transgene, *Oct4-EGFP*, in the TgOG2 line of transgenic mice is restricted to the germ line; furthermore, peripheral blood cells expressing the panleukocyte marker, CD45, expressed neither EGFP nor germ cell markers. However, those cells designated as oocytes were not examined for haematopoietic markers *in situ*, which analysis would have been definitive. The hypothesis of Eggan *et al. *[14] is developed further here, by considering the possible involvement of one particular CD45^+ ^and SSEA1^+ ^cell type, the macrophage, which is a differentiated derivative of circulating monocytes. Inspection of photomicrographs presented by Tilly *et al. *[15] as depicting *de novo *oocytes in follicular nests reveals centrally within those nests large, non-spherical (and EGFP positive) cells with irregular nuclei, cytoplasmic inclusions and numerous, clear cytoplasmic vacuoles (see Figure one, right-hand panel, in Tilly *et al. *[15]): these features are highly reminiscent of macrophages rather than oocytes. Figure two ' B' in Lee *et al. *[13] shows a similar EGFP-positive cell within a follicle, dissimilar in morphology to an oocyte, with cytoplasmic inclusions resembling phagocytised granulosa cells (one of which appears to be membrane enclosed). Johnson *et al. *[12] contend that their female germline stem cells express SSEA1. However, in addition to its status as a classical, murine stem cell marker, SSEA-1 is a haematopoietic differentiation antigen expressed on most terminally differentiated myeloid cells.

Crucially, the identification of oocytes from co-expression of germ-cell markers with EGFP immunofluorescence in experiments using the TgOG2 mouse [12,13] rests on the exclusivity of expression of *Oct4-EGFP *in the germline. However, Yoshimizu *et al. *[90] reported that in TgOG2 transgenic embryos, EGFP expression is not entirely germ-cell specific, with "faint but significant expression" throughout the epiblast. (This observation was analysed further and attributed to the presence of residual elements in the epiblast-specific enhancer [56].) Moreover, the original analysis of tissue-specific expression in adult TgOG2 mice [91] was not exhaustive. It is relevant that expression of *Oct4 *has been reported in adult stem cell populations and tumours [58,92], human diseased arteries [93], and rabbit atherosclerotic plaques [94], by unknown regulatory mechanisms. The hypoxia-inducible factor, HIF-2α, has been shown to bind directly to the *Oct4 *promoter and enhancer regions, activating the gene and eliciting a tumorigenic activity [95]. Therefore, can *Oct4 *transcription from the distal enhancer be considered as absolutely germ-cell specific? A factor present in *Xenopus *oocytes, tumour-associated factor or Tpt1, activates *Oct4 *transcription in mouse somatic-cell and ES-cell nuclei by binding to the *Oct4 *gene sequence directly - effectively bypassing the promoter and enhancer elements [96]. Tpt1 is expressed by macrophages resident in the testes of neonatal and adult male rats, and in adult human testis [97]. Therefore, it is suggested that macrophages have the inherent capacity, through expression of Tpt1, to transcribe embryonic forms of *Oct4*.

Lee *et al. *[13] derived mononuclear cells from peripheral blood of TgOG2 female mice, and were unable to detect EGFP^+ ^cells in the CD45^+ ^fraction. Therefore it is inferred here that *Oct4-EGFP *expression may occur in macrophages, but not the circulating monocytes from which the tissue macrophages derive. Expression of *Oct4 *by the macrophage has been reported, in atherosclerotic plaques of rabbits [94].

#### (b) Potential involvement of the macrophage

A further reason to implicate the macrophage in the structures identified as *de novo *oocytes [12,13] arises from the various functions it performs in the ovary [98]. The macrophage has been documented within atretic follicles [99], where it clears apoptotic granulosa cells. In the foetal pig ovary, macrophages have been observed to phagocytise degenerating oogonia and oocytes, the nuclei being clearly visible in the macrophage cytoplasm [100]. Pepling and Spradling [33] have shown that apoptotic oogonia still demonstrate Mvh antigenicity. Therefore, could some designated oocytes (e.g. Figure seven 'M'-'O' in Johnson *et al. *[12]) that co-express oocyte markers and EGFP consist of macrophages performing phagocytosis of an oocyte? The phenomenon interpreted as *de novo *oocytes [12,13,15] therefore might be explained by macrophage clearance of degenerating and/or apoptotic oocytes following ovotoxic treatment, by phagocytosis and antigen processing. This hypothesis predicts that the structures in question would arise more rarely during homeostasis and parabiosis than following ovotoxic treatment; and that the timing of detection is crucial, the clearance of degenerating oocytes occurring over weeks. This may explain why EGFP-labelled structures can be detected within 30 h of transplantation [12], and yet show variable detection after 2 months (Eggan *et al. *[14]* versus *Lee *et al. *[13]). There emerges a need for *in situ *analysis using markers for immune cells, as advocated by Eggan *et al. *[14], in order to test these possibilities.

#### (c) *De novo *oocytes as potential artefacts

Johnson *et al. *[12] transplanted peripheral blood cells from *Oct4-EGFP*-carrying TgOG2 mice to CT-treated wt and *Atm*^-/- ^female mice, to establish migration of blood-borne oocyte precursors to the depleted ovary. The authors presented photomicrographs (Figure seven, 'A'-'R') in which presumptive *de novo *oocytes in non-follicular structures stain positively by immunofluorescence for EGFP and germ-cell markers. However, the aspect of images 'A'-'L' and 'P'-'R' resembles autofluorescence - indeed, the artefact was indicated by the authors in neighbouring cells in Figure seven, 'P'-'R'. Autofluorescent cells include macrophages, dendritic cells, lymphocytes and granulocytes. The designated oocytes in Figure seven, 'A'-'L' and 'P'-'R', resemble dendritic cells, which are highly fluorescent and emit within the wavelength spectrum of the fluorochromes, fluorescein, isothiocyanate and phycoerythrin [101]. Autofluorescence has been reported previously for luteal cells of the macaque [102], and stromal tissues of the rat ovary [103].

#### (d) Distinct temporal and spatial niches for germ cell and haematopoietic lineage specification

Finally, in considering a possible supply of extra-ovarian germ cell precursors, Johnson *et al. *[12] reasoned that the bone marrow would be a logical source, due to a stated similarity in location and timing of embryonic haematopoietic induction and PGC specification. As with the PGC, segregation of the haemangioblast, the precursor of haematopoietic and endothelial lineages, occurs in a temporally and spatially defined manner. It is a mesodermal derivative of transient existence, arising within the length of the posterior primitive streak during a 12-18 h window, from midgastrulation (E7) to head-fold stages. Haemangioblasts differentiate rapidly on emigration from this origin [104] towards two sites: the yolk sac, for the primitive erythroid lineage, and endothelial and vascular smooth muscle progenitors; and the para-aortic splanchnopleura, for lymphoid progenitors and HSC. Therefore, the PGC and haemangioblast differ in their site of emergence (base of the allantois, *versus *a more distal location in the posterior primitive streak, respectively), and in their immediate progenitors (proximal and posterior epiblast, *versus *mesoderm). The exact location of PGC and of haemangioblast derivatives within the extraembryonic tissues also differs (base of the within extraembryonic mesoderm, *versus *on the yolk sac surface facing the exocoelomic cavity, respectively, by E7.5). Furthermore, ectopic PGC have only been observed in the mesonephric tissue, where they undergo meiotic arrest [105]. No PGC have ever been noted in the circulation of mammals [106]. Moreover, the gene expression profile of germ cells from precursor stages to PGC specification is lineage specific, with sequential induction *Blimp1 *[107], *Fragilis *and *Stella *[108], and down regulation of somatically expressed genes. Therefore there is no evidence for a separate or branching germline during gastrulation.

It should also be emphasised that to date, no definitive evidence exists that those oocytes that are recruited for maturation and fertilisation *in vivo *originate from any other source than the classical germline. Furthermore, the ovary remains the exclusive site of regulation of meiosis and oocyte maturation.

### (v) Functional, female germline stem cells

**Figure 1:**
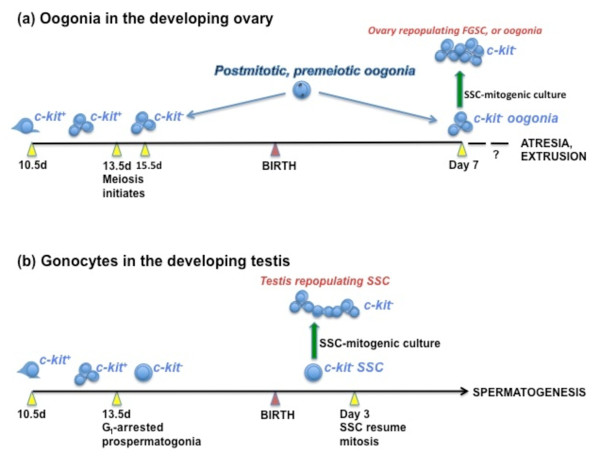
**Proposed origin of FGSC from residual oogonia in the neonatal mouse ovary**. During embryogenesis, PGC colonise the genital ridges at 10-11 d.p.c., transforming into (a) oogonia in the developing ovary, or (b) gonocytes in the developing testis. Both phenotypes undergo clonal expansion within syncytia until ~13.5 d.p.c., when proliferation ceases concurrently with downregulation of *c-kit *expression [121]. In (a), a minority of oogonia within germline cysts enter meiosis, while the majority arrest and eventually undergo apoptosis [33]. By 15.5 d.p.c., *c-kit *expression is undetectable in oogonia, indicating universal growth arrest [121,123]. A proportion of oogonia persist in germline cysts after birth [109], comprising 10% of germ cells at day 7 postnatal [33]. The postnatal survival period of germline cysts is unknown. It is hypothesised that the residual oogonia occupy postmitotic and premeiotic stages of the cell cycle up to preleptotene, denoted here by an oogonium with condensed chromatin peripheral to the nuclear membrane. The preleptotene stage was described previously as a control point for entry into meiosis and G_1 _arrest [147], and also for relapse into mitosis [149,150]. (In *S. cerevisiae*, reversion to mitosis has been demonstrated during meiotic differentiation, even after premeiotic DNA synthesis [151]). Therefore, postmitotic oogonia isolated from neonatal ovaries may resume division under conditions that stimulate SSC to proliferate as gonocytes [114,148], while the oogonial phenotype and capacity for *in vivo *folliculogenesis [115] are maintained. This is the proposed origin of reported FGSC lines [10]. Similarly, residual oogonia may constitute the oocyte-producing component of cultures obtained by Pacchiarotti *et al. *[11] using SSC-based conditions [63]. In (b), gonocytes arrest in G_1 _as prospermatogonia (large interphase nucleus) at 13.5 d.p.c., resume mitosis at day 3 postnatal, and enter meiosis at day 7 postnatal. Absence of *c-kit *expression is depicted as a diagnostic feature of postmitotic oogonia and prospermatogonia [121,123], which is shared by FGSC [10] and SSC [114] lines.

Another challenge to the concept of a fixed ovarian pool at birth was made by Zou *et al. *[10], who claimed to have isolated female germline stem cell (FGSC) lines from both neonatal and adult mice ovaries (the adult mice being of unspecified age), having first identified putative FGSC in the OSE of neonatal and adult mice by BrdU-incorporation (see section **(i)**, above). Remarkably, FGSC lines were shown to be capable of reassembly into follicles on reintroduction into a sterile ovary, and produced viable offspring that transmitted a transgene through the germline. The authors take their considerable achievements as validating the existence of a germline stem cell population in the ovary, but do not consider the possibility that their lines arise from quiescent oogonia present in the postnatal ovary, which are induced to proliferate in culture under conditions devised originally to be highly mitogenic for SSC (Figure 1). Arguments leading to this conclusion are presented below. A starting premise is the existence of oogonia in the postnatal mouse ovary, as documented previously by Pepling and Spradling [33], and Greenbaum *et al. *[109]: about 10% of germ cells persist within small germline cysts containing 2-4 cells at 26.5 d.p.c., or day 7 postnatal [33].

#### (a) Constituent phenotypes of explanted germ cells include oogonia

A relatively straightforward procedure was used by Zou *et al. *[10] to isolate FGSC lines: cell suspensions were prepared from whole ovaries, and a very few cells (approximately 10 per mouse) were isolated by immunomagnetic separation using anti-Mvh antibody. Although the location of Mvh is usually considered to be cytoplasmic in PGC, oogonia and oocytes [41], the stated rationale for this separation was based on the presence of purported trans-membrane sequences in the Mvh protein [10]. The validity of these sequence assignations was questioned by Abban and Johnson [16], who emphasised the need for further analysis of FGSC surface immunogenicity. It may be relevant, in this connection, that specific Fc receptors, Fc_γ _R_I, II, III_, are present on oocytes [110-112], and an IgG-binding antigen has been demonstrated in SSC [82]. Therefore the possibility arises that in the study of Zou *et al. *[10], cell isolation resulted from an artefact of the antibody coated microbeads binding *via *their Fc moieties to the Fc receptors [113] on the oolemma, if not also on the plasma membrane of the oogonia, the female counterparts of SSC (which theme is developed below). According to conventional theory [1], the purified, Mvh-expressing germ cells should consist entirely of (ZP-free) primary oocytes and oogonia, without contribution from any distinct population of germline stem cells.

#### (b) The morphology of FGSC lines resembles that of cultured oogonia

In the system of Zou *et al. *[10], cells proliferated in a feeder-based culture system formulated initially for SSC expansion, containing LIF, putrescine, EGF, GDNF, bFGF, insulin and transferrin. The proliferating cells that resulted were described as forming compact clusters and having blurred cell boundaries - these are characteristic features of oogonia proliferating in ovarian germline cysts [33], as well as proliferating SSC [114]. The morphology of FGSC in culture also resembles that of cultured oogonia (which in some earlier publications are referred to as mitotic PGC having reached the non-motile phase) [115-119]: namely, rounded cells with large nuclei and without lamellipodia, with moderate alkaline phosphatase staining, and non-adherent to the substratum. In culture, the (earlier, migratory phase) PGC proper transform with time into cells having this morphology [117].

Previously the long-term culture of oogonia was problematical. The inability to extend the culture period substantially was attributed to the cell-autonomous behaviour of PGC and their derivatives, causing growth arrest as well as morphological changes. Kawase *et al. *[116] and Nakatsuji *et al. *[118] prolonged proliferation to a limited degree by specific culture conditions or suppression of apoptosis, respectively.

#### (e) Cultured oogonia undergo development and ovulation *in vivo*

Previous studies have demonstrated the ability of cultured oogonia to assemble into follicles when recombined with ovarian somatic cells [66,119], and to produce live offspring on transplantation into partially ovariectomised mice [115].

#### (f) The gene expression profile of FGSC resembles that of growth-arrested oogonia

Zou *et al. *[10] noted that their FGSC lines are dissimilar to ES cells in their gene expression pattern: FGSC expressed *Oct4*, *MVH*, *Dazl*, *Blimp-1*, *Fragilis*, *Stella *and *Rex-1*; but not *c-kit, Figla *(a marker for primordial follicle formation), *Sox-2*, *Nanog*, *Scp1-3 *or *ZP3*. The combined expression of *Oct4, Sox-2 *and *Nanog*, the regulatory network of genes for maintaining multipotency, is considered prerequisite to a self-renewing stem cell population, not only in embryonic but also in adult systems [58] (see also section **2**.**(ii)**). Therefore, the FGSC expression pattern is inconsistent with a stem-cell phenotype, specifically mouse EG cells and mouse PGC, which are *Sox2*^+^, *Nanog*^+^, *c-kit*^+^[65]. However, the gene expression profile of FGSC (*Oct4*^+^, *MVH*^+^, *Dazl*^+^, *Blimp-1*^+^, *Fragilis*^+^, *Stella*^+ ^and *Rex-1*^+^; *c-kit*^-^, *Figla*^-^, *Sox-2*^-^, *Nanog*^-^, *Scp1-3*^- ^and *ZP3*^-^) is more consistent with oogonia [3,41,64,65,120] except for one notable feature - a lack of *c-kit *expression. During development of male and female mouse germ cells, *c-kit *expression ceases coincident with entry into the non-proliferative phase, between 13.5 and 15.5 d.p.c. [121,122]; and *c-kit *expression is absent from oogonia at 15.5 d.p.c. [123]. Therefore the possibility arises that the founding population of cells giving rise to the FGSC lines of Zou *et al. *[10] are growth-arrested oogonia, proposed to reside within those residual, small cysts of the neonatal mouse ovary [33,109]. Oogonia, like FGSC, are diploid and carry erased, gynogenetic imprints [3,120].

#### (g) Functional parallels between FGSC and SSC

It is significant that the culture medium used to derive the FGSC lines was used initially for the derivation of SSC lines [10]. In the adult mouse testis, *c-kit *is expressed by differentiating spermatogonia, but not by undifferentiated, testis repopulating SSC [63,81]. *C-kit *expression was analysed in the first SSC lines to be isolated [114], and found to be absent from undifferentiated, proliferating SSC and confined to differentiating derivatives.

Therefore, the capacity of oogonia to proliferate (without resumption of *c-kit *expression) in medium optimised for SSC would provide an additional example of the sex-independent properties of male and female germ cells up to the stage of growth arrest [122,124]. To paraphrase Baltus *et al. *[124], premeiotic DNA replication is a terminal differentiating event in the oogonium as a sexually undifferentiated precursor cell. By extrapolation of this insight, the premeiotic oogonia in the postnatal ovary have not yet undergone the differentiation process, and may be prone to resume mitosis provided that specific culture requirements are met. This hypothesis is developed in the legend to Figure 1.

#### (g) Implications of lack of *c-kit *expression by FGSC and oogonia

A predicted consequence of the lack of *c-kit *expression by FGSC of Zou *et al. *[10] is resistance to the effects of BU (as in SSC, see section **(iii)**). Therefore, BU administration should not eliminate this purported stem-cell population *in vivo *and oogenesis should resume with time, as is observed for spermatogenesis in the BU-treated male mouse [81]. However as noted in section **(iii)**, the converse is observed as BU treatment leads to extinction of female fertility [7,80]. This provides circumstantial evidence against FGSC acting as facultative stem cells to support *neo-*oogenesis *in vivo*, either during homeostasis or following ovotoxic damage. By the same rationale, the lack of *c-kit *expression by growth-arrested oogonia [121-123] argues against their status as functional stem cell progenitors of ooyctes *in vivo*.

Nevertheless it is of interest to establish the size and cell cycle status of the oogonial population in the post-natal ovary. However, persistence of mitotic oogonia in the adult mouse is difficult to reconcile with the absence of detectable SSEA-1 in germ cells of the adult ovary [17], because oogonia proliferating *in vivo *are positive for this marker [66]. In the human, clusters of residual oogonia have been noted in late foetal ovaries but never in adult ovaries; and were thought to arise from errors in follicular development, and to be destined for elimination [125]. That the same fate (apoptosis, extrusion) applies ultimately to those residual, growth-arrested oogonia in the neonatal mouse ovary is favoured here.

## 3. *Neo*-oogenesis *versus *classical theory: accounting for fertility preservation, post CT-induced ovarian failure, by BMT

So far, alternative explanations have been presented for main observations advanced in support of *neo-*oogenesis, leading to the proposition that the hypothesis is erroneous and may lead to false directions for the preservation of female fertility. This is illustrated by a significant observation made by Johnson *et al. *[12] and Lee *et al. *[13] in adult female mice subjected to ovotoxic CT: BMT to these mice resulted in restoration of follicle production, compared with continued sterility in CT-treated mice not receiving BMT. Johnson *et al. *[12] and Lee *et al. *[13] regard this observation as validating their contention that a reservoir of germline stem cells exists in the bone marrow, so that BMT reinstates host *neo-*oogenesis by delivery of oocyte precursors. However, the observation of Johnson *et al. *[12] and Lee *et al. *[13] may be interpreted differently, in accordance with a fixed oocyte reserve [1]. This alternative explanation draws on currently proposed mechanisms of autoimmune ovarian failure to suggest a protective effect of BMT on resident oocytes, which possibility previously was discounted [13].

**Figure 2:**
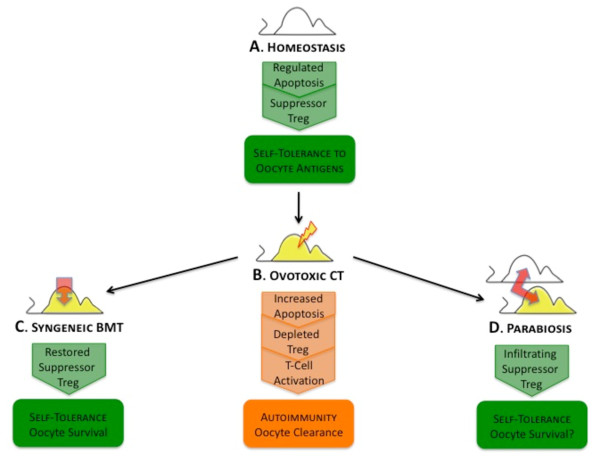
**Hypothesis for the restoration of fertility in CT-treated female mice following BMT**. (A) Self-tolerance to ovarian antigens during homeostasis. In the steady-state ovary, antigens produced by apoptotic oocytes in atretic follicles [126] constitutively stimulate Treg to suppress an autoreactive T cell response and maintain self-tolerance [127,128]. Thus, low-level apoptosis protects against autoimmunity [131]. (B) Ovotoxic CT precipitates autoimmunity *via *increased apoptosis and CY-induced Treg depletion. The ovotoxic CT combination of CY and BU [12,13] enhances oocyte apoptosis and antigen release, promoting autoimmunity [131]. Moreover, specific effects of CY, namely augmentation of effector T-cell stimulation and reduction of Treg numbers and function [132], stimulate autoimmunity to ovarian antigens. A proportion of oocytes may survive CT-induced damage, but the switch to autoreactivity causes their immune clearance and ovarian failure. (C) Restoration of self-tolerance by BMT. In the mouse with developing autoimmunity to ovarian antigens caused by CT, suppressive Treg function - and therefore self-tolerance - is restored by haematopoietic chimaerism following syngeneic BMT. Consequently, any undamaged primordial follicles avoid immune clearance, sustaining fertility. That beneficial effects on fertility are absent when BMT is postponed from 1 week to 2 months following CT [13] accords with a temporal window for donor Treg to suppress autoimmunity efficiently, beyond which an autoreactive T cell response predominates [132]. (D) Parabiosis. This hypothesis predicts that in the parabiotic system of Eggan *et al. *[14], where CT-treated female mice were connected to untreated partners (1 d later) by their circulations, priming of an ovarian autoreactive T cell response in the CT-treated mouse would be suppressed by functional Treg infiltrating from the untreated mouse, thereby imposing dominant self-tolerance in both parabionts. The use of superovulation to measure ovarian function [14] may have precluded detection of restoration of fertility in CT-treated mice by parabiosis, and in CT-treated (nonparabiotic) mice by BMT (see section 3.(D)).

A series of events is posited to occur during the experimental manipulations [12,13], illustrated in Figure 2: **(A) **maintenance of self-tolerance to ovarian antigens during homeostasis; **(B) **after ovotoxic CT, induction of apoptosis in follicular cells leading to failure of tolerance, induction of autoimmunity against ovarian antigens, and subsequent destruction of surviving follicles; and **(C) **after BMT and establishment of haematopoietic chimaerism, restoration of tolerance and resumption of development of surviving follicles. Evidence for these events is now considered in detail.

### (A) Self tolerance in the steady state ovary

It has been amply demonstrated in mouse models that during homeostasis there predominates self-tolerance to ovarian antigens, sustained by a mechanism dependent specifically on regulatory T cells (Treg). (Treg, either thymus derived or produced by activation of naïve T cells, function to suppress activation of T, B and NK cells.) In the steady-state ovary, atretic follicles contain degenerating ooyctes that are the targets of autoreactive CD4^+ ^T cells [126]. Concurrently, ovarian antigens continuously stimulate Treg in the regional lymph nodes to maintain self-tolerance [127,128]. Autoimmune ovarian disease (AOD) results from loss of functional Treg, e.g. by thymectomy or iatrogenic effects, so that self-tolerance is converted into an active T cell response [128-130]. In their study of AOD in the thymectomised mouse model, Wheeler *et al. *[128] further demonstrated that ovarian antigen-specific Treg are capacitated by autoantigen exposure within regional, draining lymph nodes; and that AOD development can be abrogated in the thymectomised recipients by transfer of Treg from the lymph nodes of normal female donors. This Treg-based mechanism may be directly relevant to the studies under consideration here [12,13].

### (B) Effects of CT treatment - induction of autoimmunity

The CT combination of cyclophosphamide (CY) and BU causes catastrophic damage to oocytes and ovarian failure [12,13], and is likely to increase apoptosis, which functions normally to promote oocyte clearance and tissue remodelling. According to current thinking, efficient apoptosis provides a safeguard against autoimmunity. But a high burden of apoptosis is strongly implicated in the development of the autoimmune state, by cellular spillage or increased exposure of the immune system to autoantigens [131]. By this reasoning, CT would serve as a trigger for autoimmunity in the ovary, whereby the load of apoptotic cells may exceed the clearance capacity of macrophages and/or dendritic cells.

Cyclophosphamide (CY), the chemotherapeutic alkylating agent used in combination with BU as an ovotoxic agent [12,13], constitutes possibly an additional trigger for autoimmunity. CY has established immune-enhancing effects, involving both the stimulatory and suppressive arms of adaptive immunity: CY treatment augments effector T-cell stimulation, while selectively depleting Treg numbers and function [132]. These actions of CY are achieved by alteration of subsets of dendritic cells in lymphoid tissue, which normally maintain peripheral tolerance *via *Treg activation [133].

In a highly relevant study that used the non-obese diabetic (NOD) mouse model, Brode *et al. *[132] demonstrated the potential of Treg to abrogate organ-specific autoimmunity, and deduced the existence of a temporal window between disease induction and development of an autoreactive T cell response during which suppression would be effective. A single injection of CY induced onset of the autoimmune syndrome, type 1 diabetes (T1D), which was synchronous with selective reduction in Treg in lymph nodes through apoptosis, and with reduced suppressive capacity of Treg *in vitro*. Furthermore, the ensuing autoreactive T cell response could be suppressed, and development of T1D abrogated, by transfer of antigen-specific Treg from a non-diabetic, syngeneic donor to CY-treated, NOD recipients - provided that Treg were received between 1 and 8 d after CY treatment. Thereafter, the developing autoreactive T cell response predominated. A mechanism was proposed for the action of CY, whereby an imbalance created between CD4^+^CD25^- ^T cells and Treg leads to priming of autoreactive T cells and development of autoimmunity. (Normally, interaction of Treg with dendritic cells within lymph nodes suppresses the priming of naïve autoreactive T cells.)

A mechanism is therefore proposed from **(A) **and **(B) **for the studies considered [12,13], by which CT treatment induced ovarian failure and apoptosis, and caused depletion of ovarian antigen-specific Treg, thereby promoting activation of effector T cells and inducing autoimmunity to ovarian antigens. A proportion of oocytes may have survived, but a switch from self-tolerance to intolerance caused these to be eventually cleared. It is in keeping with this hypothesis that autoimmune premature ovarian failure in humans is characterised by inflammatory infiltration into developing follicles and the production of anti-ovarian autoantibodies, while primordial follicles are spared [134-136]. Also, immunosuppressive, corticosteroid therapy may lead to resumption of menses in women with autoimmune oophoritis with secondary amenorrhea [136].

### (C) Restoration of tolerance

In mouse models, reconstitution of the immuno-haematopoietic system by BMT or transfer of HSC attenuates autoimmunity and may achieve disease remission (reviewed by Kaminitz *et al. *[137]). The mechanism of such modulation may involve resetting immune homeostasis [138-140] or reversal of spontaneous autoimmunity, for example by clonal deletion, anergy, or suppression [137,141]. In the proposed scenario of the ovary with developing autoimmunity to ovarian antigens following CT **(A, B)**, immuno-modulation would be restored by the haematopoietic chimaerism induced by syngeneic BMT, with resumption of self-tolerance. And it is suggested that the specific tolerogenic mechanism would involve restoration of suppressive Treg function by BMT, either from donor stem cells developing in the recipient's thymus, or from bone marrow acting as a natural reservoir for homeostatic trafficking of functional, activated Treg [142]. The transient immunosuppressive and lymphopenic effect of CY [143], given in combination with BU as ovotoxic treatment, also would provide a niche for homeostatic expansion of Treg following BMT [130]. Consequently, remaining primordial follicles would grow and reach ovulation, rather than be cleared as in control, CT-treated mice not receiving BMT.

Returning to the study of Lee *et al. *[13], several observations can now be reinterpreted:

**(i) **Beneficial effects on fertility are attenuated when BMT is postponed from 1 week to 2 months following CT. This can be explained by the priming of an autoreactive T cell response after 1 week, in accord with Brode *et al. *[132] (see **(B)**, above). BMT at 2 months is ineffective, and there is progressive destruction of the surviving oocyte reserve by immune clearance.

**(ii) **Postponement of mating after CT and BMT by two months *versus *1 week results in decreased fertility. This can be explained by exhaustion of the surviving oocyte reserve in the 2-month interim period by oestrus cycling (occurring every 3-5 days), compared with the suspension of oestrus cycling during consecutive pregnancies.

This line of reasoning also may account for the observations of Johnson *et al. *[12] with *Atm*^-/- ^mice. Although ovaries of mutant females are described as devoid of oocytes and developing follicles [12], evidence exists for the persistence of residual germ cells: rare and abnormal oocytes were recorded in *Atm*^-/- ^mice that were 20 days old [144], and between 17 and 29 days old [145]. This accords with the observation of Johnson *et al. *[12] of low-level, germ cell-specific gene expression (*Oct4*, *Mvh*, *Dazl *and *Stella*) in ovaries from adult *Atm*^-/- ^mice. Crucially, Johnson *et al. *[12] noted that, following ovotoxic CT and BMT, ovaries from *Atm*^-/- ^mice contained a small number (maximum, 25) of follicles at 2 and 11.5 months after BMT, while non-transplanted mice did not. It is suggested here that ovarian failure caused by *Atm*-deficiency also may induce autoimmunity to ovarian antigens, resulting in clearance of those rare, residual oocytes. Thus, BMT to *Atm*^-/- ^mice would restore tolerance, allowing those surviving oocytes to develop to antral stages [12].

This hypothesis of CT-induced autoimmunity and its suppression by immune-cell transfer gives a prediction for the system of Eggan *et al. *[14], where female mice were subjected to CT (with BU and CY) and 1 d later were connected parabiotically to untreated partners, to provide a shared circulatory system. In this case, the priming of an ovarian autoreactive T cell response in the CT-treated mouse would be suppressed by functional, ovarian antigen-specific Treg transfusing from the untreated partner, thereby imposing a state of dominant self-tolerance in both parabionts (Figure 2D). That Eggan *et al. *[14] found no evidence for restoration of fertility in the CT-treated mice by parabiosis, nor in CT-treated (non-parabiotic) mice by BMT, most likely reflects their use of superovulation as the measure of ovarian function. (The technique was used primarily to harvest large numbers of oocytes, to ascertain any contribution of blood-derived precursors to *neo-*oogenesis.) However, there is inherent variance in the superovulatory response in mice (which is apparent in the data presented), and a dependence of superovulation on the instantaneous number of hormonally responsive, antral follicles. Consequently, the superovulated yield would not reflect the size of the primordial and growing follicle pool - especially when these are close to exhaustion following CT. Therefore, superovulation would not provide an accurate indicator of long-term fertility. The measures taken by Johnson *et al. *[12] and Lee *et al. *[13] to assess fertility are more effective, analysing total numbers of non-atretic immature follicles per ovary, and recording live-birth pregnancies over time, respectively.

To paraphrase Oktay and Oktem [146], further investigation is needed into the mechanism of rescue of fertility in CT treated females by BMT. Establishing the validity or otherwise of the *neo-*oogenesis concept is crucial to understanding germinal function, and to preserving fertility. The proposed involvement of the immune system may, on the other hand, offer possibilities for preserving ovarian function in women undergoing CT, and for treatment of AOD and primary ovarian insufficiency, e.g. by Treg-based immunotherapy [130].

## 4. Conclusions

In summary, re-examination of experimental findings cited by proponents of *neo-*oogenesis in mammals as validating their hypothesis leads to alternative interpretations drawn from published literature, which are entirely consistent with the long-standing orthodoxy of a determinate oocyte reserve [1]. By comparing those studies collectively advocating *neo-*oogenesis, recurrent themes emerge.

Firstly, several studies used as starting material ovaries from mice either termed 'juvenile' [7] or specified as day 5 [10] or days 2-5 postnatal [11], to locate mitotic germline stem cells within the OSE. This may represent an injudicious choice as the neonatal period between birth and day 5 postnatal is one of flux for mouse ovarian germ cells: at this time, ovarian cyst breakdown occurs concomitantly with a high rate of oocyte attrition, while meiotic progression continues in other oocytes towards diplotene with formation of primordial follicles [41]. That the majority of oocytes attain diplotene only by day 5 postnatal signifies that meiotic DNA recombination and repair may still be ongoing in tardy oocytes, which phenomenon is argued to underlie the observation of BrdU incorporation into germ cells, as well as apparent mtDNA synthesis (section **2**.**(i)**). (Regarding BrdU incorporation by germ cells in adult ovaries [10], the precise extent of DNA recombination/repair in oocytes at later stages is unknown.) Added to this, oocytes that are defective or delayed may be actively extruded from the OSE [45] (and see section **2**.**(i)**). In short, the aforementioned studies involving *in situ *anti-BrdU immunocytochemical analyses of germ cells at the surface of the neonatal ovary [7,10,11] may have captured these dynamic processes, rather than mitotic replication of germline stem cells. Crucially, in the neonatal ovary a significant proportion (~10%) of germ cells persist in germline cysts [33], which are posited here to provide the founding cell type for the FGSC lines of Zou *et al. *[10], and to be a likely component of the cultures produced by Pacchiarotti *et al. *[11]. The potential involvement of oogonia in those cultures of putative germline stem cells is a second recurring theme.

A third theme is misidentification of somatic cell types as germ cells (section **2**.**(ii)**). It is inferred that cultures of putative germline stem cells derived from mouse ovaries [8,9,11] were confounded by the presence of somatic cells, from cell morphology and gene expression patterns. In some cases cell lines were identifiable as regenerative ovarian epithelium [9,11]. This cautions against reliance on presumed germ-cell specificity of markers for stem-cell validation, and emphasises the need for recognition of OSE as a complex and multipotent tissue [52]. Concerns for cellular misidentification extend also to immune cells of the ovary, and specifically macrophages, which are suggested instead to constitute those structures described as *de novo *oocytes provided by blood-borne precursors [12,13,15].

A fourth emerging theme is that detailed consideration of the c-kit/SCF signaling pathway in germ cells, in the light of data presented [7,10], countermands the existence of *neo*-oogenesis and female germline stem cells. Much has been made of the sterilising effects of BU treatment in female mice as proving the existence of germline stem cells in the ovary [7,12,15]; but section **2**.**(iii) **provides arguments directly contradicting statements by Tilly *et al. *[15] that BU targets only replicative stem cells, and not primordial or later follicles. Data from spermatogenic and haematopoietic systems, where the respective stem cells are refractory to the effects of this agent, indicate that abrogation of c-kit function is central to its mechanism of toxicity [81]. This both predicts and explains the observed, devastating effects of BU on folliculogenesis, from primordial to later stages, where both germ and somatic cells depend on functional c-kit/SCF signaling for survival. Observations that BU dosages causing transient myelosuppression [87] produce irreversible sterility in female mice [7,80] are therefore consistent with a fixed oocyte reserve, without a stem cell compartment, and without replenishment from bone marrow-derived precursors. Furthermore, the proliferating FGSC of Zou *et al. *[10] lack *c-kit *expression, and therefore would be refractory to BU treatment *in vivo*. That fertility is not restored with time following BU treatment in female mice [7,80], as it is in male mice [81,82], argues against this population occupying a facultative stem cell niche *in vivo*, as was proposed previously [25,26].

The FGSC lines of Zou *et al. *[10] were equated to cultures of oogonia, from cell morphology, patterns of gene expression, functionality as oocyte precursors following transplantation to ovaries, and by comparison with previous studies. This cell type may further reinforce the functional equivalence of premeiotic male and female germ cells [124,147]; in this case with respect to growth requirements for proliferation in culture, as conditions for FGSC culture were optimised previously for SSC [10,148]. Historically, efforts were directed towards isolation of EG cells from PGC, which transformation can be achieved in germ cells isolated up to day 12.5 p.c. [3]. Therefore, the experimental utility of oogonia, so well exemplified by the work of Zou *et al. *[10], may have been overlooked in the pursuit of EG cells, until now.

Finally, an explanation was offered for the observed restoration of fertility in CT treated mice by BMT that is entirely in keeping with the orthodox theory of a fixed oocyte reserve [1], rather than *neo*-oogenesis: i.e., by regulation of autoimmunity, and not by the supply of blood-borne oocyte precursors. The clinical importance of conserving fertility in women undergoing CT gives urgency to the resolution of this ongoing controversy.

## Abbreviations

AOD: autoimmune ovarian disease; BMT: bone marrow transplantation; BrdU: 5-bromo-2-deoxyuridine; BU: busulphan; CT: chemotherapy; CY: cyclophosphamide; EGFP: enhanced green fluorescent protein; EG: embryonic germ (cell); ES: embryonic stem (cell); FGSC: female germline stem cells; HSC: haematopoietic stem cells; i.p.: intra-peritoneal (injection); MVH: mouse VASA homologue; mtDNA: mitochondrial DNA; nDNA: nuclear DNA; NOD: non-obese diabetic; OSE: ovarian surface epithelium; PBCT: peripheral blood cell transplantation; PGC: primordial germ cell; SSC: spermatogonial stem cells; T1D: type 1 diabetes; wt: wild type; ZP: zona pellucida.

## Competing interests

The author declares that she has no competing interests.
